# A risk factor attention-based model for cardiovascular disease prediction

**DOI:** 10.1186/s12859-022-04963-w

**Published:** 2022-10-14

**Authors:** Yanlong Qiu, Wei Wang, Chengkun Wu, Zhichang Zhang

**Affiliations:** 1 Institute for Quantum Information and State Key Laboratory of High Performance Computing, College of Computer Science and Technology, National University of Defense Technology, 109 Deya Road, Changsha, 410073 People’s Republic of China; 2 College of Computer Science and Engineering, Northwest Normal University, 967 Anning East Road, Lanzhou, 730070 People’s Republic of China; 3 College of Computer, National University of Defense Technology, 109 Deya Road, Changsha, 410073 People’s Republic of China; 4 National Supercomputer Center in Tianjin, 10 Xinhuan West Road, Tianjin, 300457 People’s Republic of China

**Keywords:** Chinese electronic medical record, CVD risk factors extraction, CVD prediction, Attention mechanism, Information fusion

## Abstract

**Background:**

Cardiovascular disease (CVD) is a serious disease that endangers human health and is one of the main causes of death. Therefore, using the patient’s electronic medical record (EMR) to predict CVD automatically has important application value in intelligent assisted diagnosis and treatment, and is a hot issue in intelligent medical research. However, existing methods based on natural language processing can only predict CVD according to the whole or part of the context information of EMR.

**Results:**

Given the deficiencies of the existing research on CVD prediction based on EMRs, this paper proposes a risk factor attention-based model (RFAB) to predict CVD by utilizing CVD risk factors and general EMRs text, which adopts the attention mechanism of a deep neural network to fuse the character sequence and CVD risk factors contained in EMRs text. The experimental results show that the proposed method can significantly improve the prediction performance of CVD, and the F-score reaches 0.9586, which outperforms the existing related methods.

**Conclusions:**

RFAB focuses on the key information in EMR that leads to CVD, that is, 12 risk factors. In the stage of risk factor identification and extraction, risk factors are labeled with category information and time attribute information by BiLSTM-CRF model. In the stage of CVD prediction, the information contained in risk factors and their labels is fused with the information of character sequence in EMR to predict CVD. RFAB makes well use of the fine-grained information contained in EMR, and also provides a reliable idea for predicting CVD.

## Introduction

Cardiovascular disease (CVD) is characterized by high morbidity and high mortality, which continues to plague human beings [1–3]. Data released by the World Health Organization shows that CVD causes more deaths each year than any other cause of death. Around 17.9 million people worldwide died of CVD in 2016, accounting for 31% of all deaths. According to the report of the China National CVD Research Center in 2018, the mortality rate caused by CVD ranked first in 2016, higher than cancer and other diseases, and the number of patients reached 290 million. CVD, as a chronic disease, does not obviously show corresponding characteristics in its hidden period of daily life. What worries us is that once its symptoms are manifested, the life safety of patients will be affected. Therefore, we hope to help clinicians achieve timely and rapid diagnoses by analyzing the electronic medical records (EMRs) of patients during daily physical examinations.

**Table 1:** Attributes of CVD

No.	Attributes	Description
1.	Overweight/Obesity (O2)	A diagnosis of patient overweight or obesity
2.	Hypertension	A diagnosis or history of hypertension
3.	Diabetes	A diagnosis or a history of diabetes
4.	Dyslipidemia	A diagnosis of dyslipidemia, hyperlipidemia or a history of hyperlipidemia
5.	Chronic kidney disease (CKD)	A diagnosis of CKD
6.	Atherosis	A diagnosis of atherosclerosis or atherosclerotic plaque
7.	Obstructive sleep apnea syndrome (OSAS)	A diagnosis of OSAS
8.	Smoking	Smoking or a patient history of smoking
9.	Alcohol abuse (A2)	Alcohol abuse
10.	Family history of CVD (FHCVD)	Patient has a family history of CVD or has a first-degree relative (parents, siblings, or children) who has a history of CVD
11.	Age	The age of the patient
12.	Gender	The gender of patient

CVD has become an important public health problem in China, and the need for coping strategies is imminent. From a realistic point of view, the effective information we can get about CVD in our daily life is limited. Fortunately, more and more hospitals in China have established standard EMR systems in recent years, which makes a large number of patients’ cases systematically recorded. With the rise of deep learning, the application based on the increasing EMRs has been continuously explored in the medical field [4, 5]. In particular, several studies have been conducted to predict the risk of CVD with the aim of targeting the attribute of high mortality due to CVD [6].

EMR can proactively make judgments based on the information and knowledge they have mastered, make timely and accurate prompts when individual health status needs to be adjusted, and provide optimal solutions and implementation plans. The EMR of patients with CVD contains accurate pathogenesis information. However, when we focused on the specific content of the EMR, it was found that it contained more information that was not very relevant to CVD. The information mainly involves the basic condition of the patient’s body or the declarative dialogue between the doctor and the patient. Moreover, when the information about possible CVD in a medical record text accounts for a small proportion, it will become difficult to effectively discover and utilize this information.

For the deep learning-based neural network model, these complicated sequential information not only reduces its attention to the information that may induce CVD, but also has a high possibility to reverse its prediction results. Huang et al. [7] have proposed to avoid redundant information in the text by allowing the model to have skip learning sequence information. Therefore, we intend to extract the necessary information from the EMR text by using the well-developed named entity recognition model. The key information considered, including 12 risk factors, is shown in Table 1. We can extract the risk factors and their attribute labels that may lead to CVD in the text of EMRs as the research objects of the experiment. However, although the training efficiency of the model can be improved based on the risk factor, the performance of the model is degraded. From the experimental analysis, we have realized that simply using relatively independent risk factors as a model to obtain knowledge sources is too single, which means that the contextual information of EMR texts is also indispensable. In response to this situation, we propose the risk factor attention-based (RFAB) Model, a two-layer architecture to model risk factors and the context of EMRs and fuse the information. We use the bi-directional long short-term memory (BiLSTM) as the encoder and decoder in the attention mechanism. BiLSTM can extract the information from the original EMRs so that our model can fully consider the global information in the EMRs of patients. For example, enhancing the information correlation between “hypertension” above and “controlling blood pressure” below is beneficial to the predictive performance of the model. At the same time, in the prediction model, we take the risk factors as the input of the attention mechanism decoder. In this way, the attention of the neural network can be focused on the vital information of risk factors leading to CVD. Experimental results show that the F-score reaches 0.9586, which fully demonstrates the effectiveness of our proposed method and network architecture. In summary, our contribution is four-fold, leading to the following conclusions:

- We no longer simply utilize the entire EMR as in previous related works, but use the 12 risk factors proposed by Su et al. [8] instead. This can well avoid the interference of a large amount of redundant information in EMRs on CVD prediction.
- The RFAB we propose contains two phases, first identifying risk factors, then predicting CVD based on the original EMR and risk factors, providing a meaningful and referential method for related predictive tasks.
- Our method does not simply predict CVD through risk factors. Through BiLSTM-CRF identification, not only the risk factors themselves are extracted, but also their corresponding tags with category information and time attribute information, which can consider more comprehensive information for prediction tasks.
- We use the character information of the original EMR text as the input of the encoder in the RFAB, the risk factor and its label as the decoder. The above two types of information are fused by the attention mechanism. This makes the predictive task focus on risk factors, and it can also take into account context information in the original EMR.

## Methodology

**Fig. 1:**
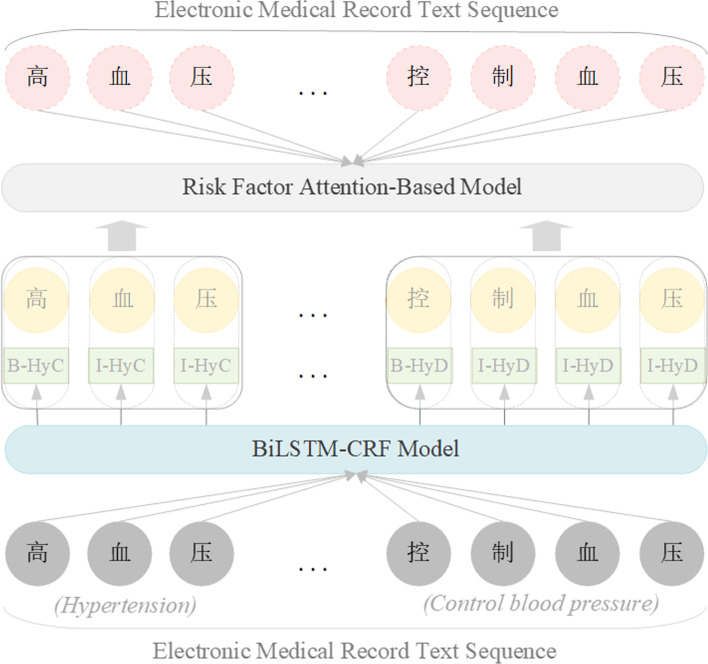
The main process of CVD prediction

The purpose of this paper is to focus on the risk factors in EMRs and to predict whether an individual suffers from CVD by machine learning methods. And the experiment is mainly divided into three stages: preprocessing the dataset, identification and extraction of risk factors, and prediction of CVD. In the data preprocessing stage, there are some missing and duplicate data in a few EMR texts, so we have carried out data cleaning and interpolation. In the stage of identifying risk factors, we use named entity recognition technology that has been widely used in industry or scientific research. The purpose is to accurately and effectively identify and extract the risk factors and their categories and time attributes in the EMRs. When we compare the recognition performance of CRF and BiLSTM-CRF, both perform well, but the latter performs better in experiments. We have analyzed the reasons in the following two aspects: On the one hand, there are many repetitions of the 12 risk factors in the EMR. On the other hand, BiLSTM is good at capturing the contextual information of text sequences, which is beneficial to identify the boundaries of entities. In the CVD prediction stage, we used the neural network model (RFAB) proposed in this paper. We present the main flow described above in Fig. 1.

### Technical details of BiLSTM-CRF model

**Fig. 2:**
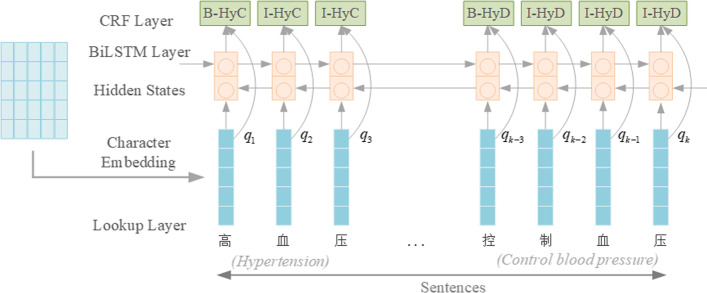
The architecture of BiLSTM-CRF model

As shown in Fig. 2, BiLSTM-CRF identifies risk factors from EMRs with the BIO (Begin, Inside, Outside) annotation scheme [9]. The labels “HyC” and “HyD” in the figure both represent risk factors for hypertension. Their temporal attributes are, respectively, that they have been with the patient (*Continue*) and during the patient’s medical treatment (*During*). In the input layer, we determine the embedding of each input character by looking up the dictionary, expressed as $Q=(q_{1},\ldots ,q_{k-3},\ldots ,q_{k})$. The character embeddings we pre-trained by the Skip-gram model [10] contain information about the words before and after it, that is, contextual information.

The model identifies risk factors by predicting the label corresponding to each character. A sequence of length *n* is inputted to the model, and the embedding layer maps characters one by one to a vector, i.e., $X=(x_{1},\ldots ,x_{t},\ldots ,x_{ n})$. Then, it is fed to the BiLSTM layer to continue encoding, and the forward and backward LSTM respectively calculate the corresponding sequence representation ${\mathop {h_{t}}\limits ^{\rightarrow }}$ and ${\mathop {h_{t}}\limits ^{\leftarrow }}$ for each character *t*. As in the LSTM memory cell implemented by Lample et al. [11], the representation of the character *t* has left and right contextual information, i.e., $h_{t}=[{\mathop {h_{t}}\limits ^{\rightarrow }};{\mathop {h_{t}}\limits ^{\leftarrow }}]$.

Then, the eigenvalues are zero-averaged by the activation function tanh, which to calculate the confidence score of the labels that each character *t* may correspond to.

1 $$\begin{aligned} e_t=\tanh (W_eh_t), \end{aligned}$$

where the weight matrix $W_{e}$ is the parameter to be learned in training.

Finally, the feature information is decoded at the CRF layer, and the best labels for characters are predicted. The *t*th column of score matrix *P* is outputted by the network correspond to the vector $e_{t}$ calculated by Eq. (1), where the element $P_{i,j}$ is the score of the *j*th tag of *i*th character in the sequence. We introduce a transition probability matrix *T* that can utilize previous annotation information when tagging the current position. $T_{y_i, y_{i+1}}$ represents the probability when tag $y_i$ moves to tag $y_{i+1}$. The optimal tags of the sequence $y=(y_{1},\ldots ,y_{t},\ldots ,y_{n})$ are obtained by solving the maximum value of Eq. (2):

2 $$\begin{aligned} s(X,y) = \sum _{i=0}^N(T_{y_i,y_{i+1}}+P_{i,y_i}), \end{aligned}$$

where the transition probability matrix will be used as a parameter of the model for training. Then, we use the softmax function to generate the conditional probability of path *y* by normalizing the scores above over all possible tag paths ${\tilde{y}}$:

3 $$\begin{aligned} p(y|X) = \frac{e^{s(X,y)}}{\sum _{{\tilde{y}}}e^s(X,{\tilde{y}})}, \end{aligned}$$

In the training process, the model predicts the best label path to obtain the highest score by computing the log probability of maximizing the correct label sequence from Eq. (4):

4 $$\begin{aligned} \arg _{ {\tilde{y}}} \max s(X,{\tilde{y}}). \end{aligned}$$

The Viterbi algorithm [12] is utilized as the dynamic programming algorithm to obtain the optimal tagging path.

### Technical details of RFAB model

**Fig. 3:**
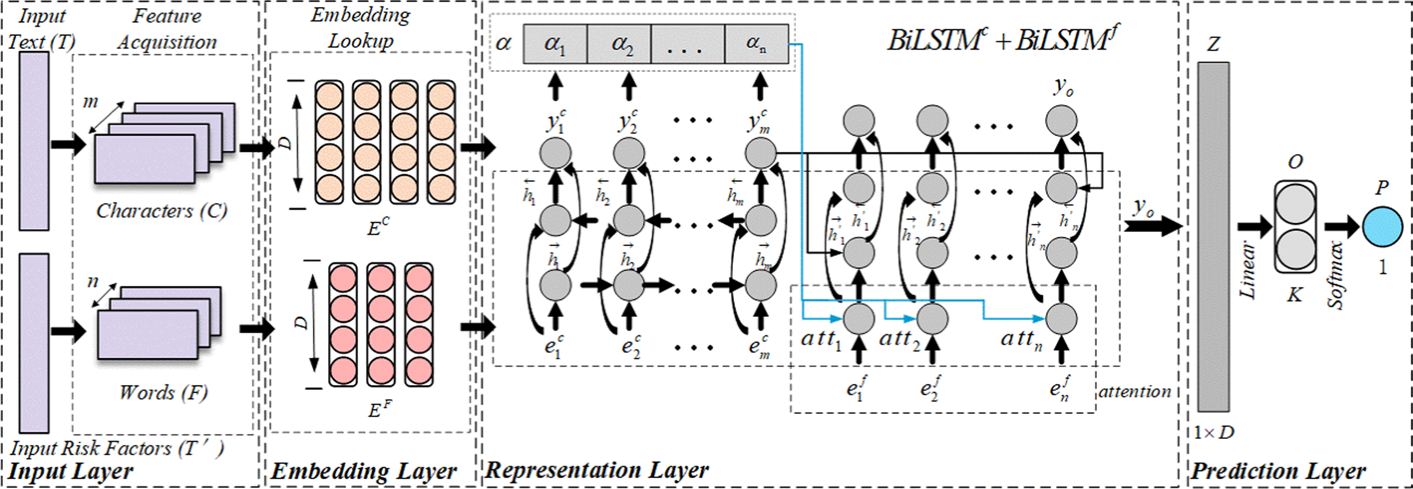
The architecture of RFAB model

As shown in Fig. 3, the purpose of our work is to comprehensively model EMRs text by using the characteristics of text content and risk factors in EMRs text, thus further realizing CVD prediction task. Generally speaking, RFAB consists of four parts: input layer, embedding layer, presentation layer, and prediction layer. The details are as follows.

*Input Layer* mainly tackles the problem of *Feature Acquisition* of the input EMR text and the input risk factors. For a Chinese raw text *T*, it contains *m* characters, i.e., $C=\{c_1,c_2,\ldots ,c_m\}$, where each character $c_i\left( 1\le i\le m\right)$ is an independent item. Meanwhile, *T* contains *n* risk factor words $W\ =\ \{w_1,w_2,\ldots ,w_n\}$, this is $T^\prime$. Since a word can often be divided into several characters, it is obvious that $n\ \le m$. Thus, the length of *C* is equal to $E^C$, and the length of *W* is equal to $E^F$, i.e., $\left| C\right| =\left| E^C\right|$, $\left| W\right| =\left| E^F\right|$.

*Embedding Layer* aims to represent each item from Input Layer in a continuous space. It accepts the characteristics of two parts of content (i.e., $E^C$, $E^F$) and outputs two embedding matrices by looking up embedding dictionary. For risk factors, we add each character-level embedding vector matched by the word correspondence, and then average to obtain the embedding vector corresponding to risk factors. As mentioned before, the lengths of the two-item features satisfy $\left| C\right| =\left| E^C\right|$ and $\left| W\right| =\left| E^F\right|$. To simplify the problem, we set the vector dimension of each of them to the same size *D*. Thus, a EMR text can be represented by two vector sequences, i.e., $E^C=\{e_1^c,e_2^c,\ldots ,e_m^c\}$, $E^F=\{e_1^f,e_2^f,\ldots ,e_n^f\}$. Exactly, these two vector sequences are also four embedding matrices, i.e., $E^C\in R^{m\times D}$ and $E^F\in R^{n\times D}$.

*Representation Layer* aims to generate a comprehensive representation of input EMR text by combining the context and risk factors information together. Corresponding to the property of character sharing, the recurrent structure of LSTM naturally processes words and characters one by one, which memorizes the characters or words that have already appeared [13]. In view of this advantage, we utilize an implementation of LSTM proposed by [14] and apply the bidirectional setting (i.e., BiLSTM) to capture both the forward and backward context information. Formally, given a specific feature embedding sequence of a sentence $s=\{x_1,x_2,\ldots ,x_N\}$, the hidden vector of a BLSTM is calculated as follows:

5 $$\begin{aligned}&\overrightarrow{h_t}\ =LSTM\left( \overrightarrow{h_{t-1}},x_t\right) , \\&\overleftarrow{h_t}\ =LSTM\left( \overleftarrow{h_{t-1}},x_t\right) , \\&y_t\ =\left[ \overrightarrow{h_t},\overleftarrow{h_t}\right] , \end{aligned}$$

where $\overrightarrow{h_t}$ and $\overleftarrow{h_t}$ is the forward hidden vector and backward hidden vector respectively at the *t*th step in the BiLSTM. And $y_t$ is the hidden output of each BiLSTM at the *t*th step, which is the concatenation of $\overrightarrow{h_t}$ and $\overleftarrow{h_t}$.

As shown in Fig. 3, there are two serialized BiLSTMs in the representation layer (i.e., $BiLSTM^c+BiLSTM^f$). In $BiLSTM^c$, the values of their initial hidden states are set to zero. Meanwhile, $BiLSTM^f$ receives the last hidden states of $BiLSTM^c$ as input, which allows the context information of characters can be further combined with the information of risk factors.

Additionally, to assign important weights to certain risk factors thus model the *risk factor sharing* property when integrating information, we design an attention mechanism which can capture the interrelations between risk factors and their corresponding Specific EMRs content. Everytime $BiLSTM^f$ receives a vector embedding of a risk factor (i.e., $e_i^f$), each $y_\epsilon ^c\in Y^c=\{y_1^c,y_2^c,\ldots ,y_m^c\}$ will conduct the dot product operation with $e_i^f$. Thus, the attention vector $\alpha ^\prime$ for $e_i^f$ is obtained as follows:

6 $$\begin{aligned} \alpha ^\prime =\left[ \alpha _1^\prime ,..,\alpha _i^\prime ,\ldots ,\alpha _n^\prime \right] ,\alpha _i^\prime =f\left( y_\epsilon ^c,e_i^f\right) ,1\le \epsilon \le m,1\le i\le n, \end{aligned}$$

where $\alpha _\epsilon ^\prime$ denote the $\epsilon$th weight of a risk factor, and $f\left( a,b\right)$ denotes the dot product function. But before the weighted sum operation, we need to normalize these weights using the softmax function, i.e., $\alpha _i$ is obtained as follows:

7 $$\begin{aligned} \alpha _i=\frac{exp\left( \alpha _n^\prime \right) }{\sum _{i=1}^{n}exp\left( \alpha _i^\prime \right) },where\sum _{1}^{n}\alpha _i=1, \end{aligned}$$

then the vector embedding of $r_i^f$ will be modified as:

8 $$\begin{aligned} \widetilde{e^f}=\sum _{i=1}^{n}\alpha _iy_\epsilon ^c, \end{aligned}$$

where $y_\epsilon ^c$ denotes the $\epsilon$th item of $Y^c$. After the attention operation (i.e., $att_i$ in Fig. 3), $\widetilde{e^f}$ have fused the weight information of risk factors. Then, $BiLSTM^f$ will further learn the contextual information of $\widetilde{e^f}$ through the calculations described in Eq. (5).

*Prediction Layer* As a result, we take the final hidden layer states of $BiLSTM^f$ (i.e., $y_o$) as the final output, and redefine it as $Z\in R^D$. Here, *Z* is exactly the ultimate representation of input EMR text *T*. After that, we feed *Z* into a fully-connected neural network to get an output vector $O\in R^K$ (*K* is the number of classes, i.e., $K=\left| U\right|$):

9 $$\begin{aligned} O=sigmoid\left( Z\times W\right) , \end{aligned}$$

where $W\in R^{D\times K}$ is the weight matrix for dimension transformation, and $sigmoid\left( \cdot \right)$ is a non-linear activation function. Finally, we apply a softmax layer to map each value in *O* to conditional probability and realize the prediction as follows:

10 $$\begin{aligned} P=argmax\left( softmax\left( O\right) \right) , \end{aligned}$$

*Model Training* Since what we are trying to solve is a prediction task, we follow the work in [15] to apply the cross-entropy loss function to train our model, and the goal is to minimize the following *Loss*:

11 $$\begin{aligned} Loss=-\sum _{T\in C o r p u s}\sum _{i=1}^{K}{p_i\left( T\right) logp_i\left( T\right) }. \end{aligned}$$

where *T* is the input EMR text, *Corpus* denotes the training corpus and *K* is the number of classes. In the training process, we apply *Adagrad* as optimizer to update the parameters of RFAB, including *W* and all parameters (weights and biases) in each BiLSTM. To avoid the overfitting problem, we apply the dropout mechanism at the end of the embedding layer.

**Fig. 4:**
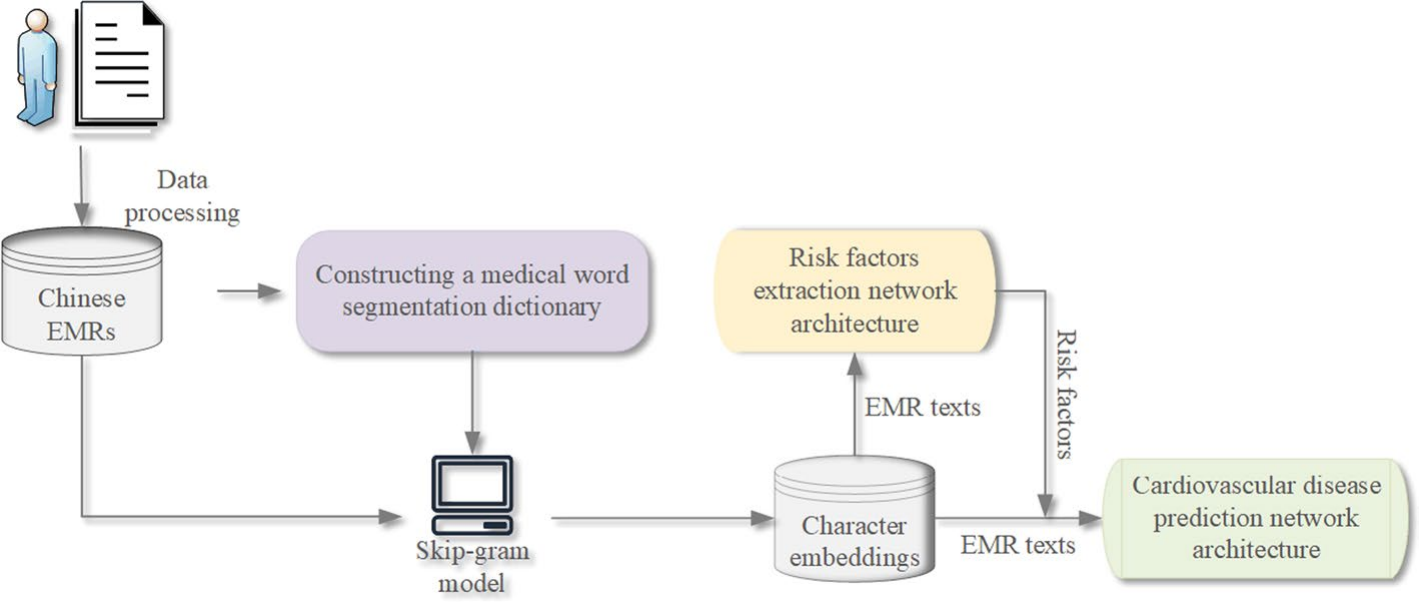
Generate the character embedding for experiments

## Results

### Dataset and evaluation metrics

The corpus involved in the experiment mainly consists of two parts: about 800,000 unlabeled EMRs and 1186 systematically labeled EMRs. The unlabeled corpus comes from the internal medicine department of a hospital in Gansu Province, and is mainly used for training and generating character-level embeddings required in the experiment process. As shown in Fig. 4, we add a risk factor dictionary during character-level embedding training. This can make the character-level embeddings trained by the skip-gram model in word2vec more relevant. Another corpus comes from the Network Intelligence Research Laboratory of Language Technology Research Center, School of Computer Science, Harbin Institute of Technology, which is mainly used to train the CVD prediction models. This corpus intends to be used to develop a risk factor information extraction system that, in turn, can be applied as a foundation for the further study of the progress of risk factors and CVD [8].

For EMRs used for CVD prediction, we need to label them as whether CVD is confirmed or not. The basis used comes from the following three parts: the first part, mainly based on the diagnosis results of clinicians in the EMR; the second part, based on the specific definition of CVD by the World Health Organization [16]; the third part, according to the first (*Symptoms*) and the third (*Diseases*) chapters of “Clinical Practical Cardiology”, an authoritative textbook for training clinicians in China, the exposition of CVD [17]. In the above three parts, the second and third parts are supplementary confirmations to the first part.

It is based on the 12 risk factors included in EMRs and their labels with category and time attributes to predict CVD, rather than directly based on the sequence information of the text of EMRs. As well as the statistics of the number of risk factors as shown in Table 2, each EMR contains multiple risk factors. From this perspective, as long as the ratio of positive and negative datasets is not seriously out of balance, we no longer suffer from the relatively small number of datasets due to the inaccessibility and legal utilization of EMRs. For the time attribute, four main types are considered in the dataset: always accompanying the patient (*Continue*); during the patient’s medical treatment (*During*); after the patient’s medical treatment (*After*); before the patient’s medical treatment (*After*). Since the *Age* and *Gender* of the risk factors do not have a time attribute, we added a time attribute as: *None*.

**Table 2:** Distribution of CVD risk factors and their occurrence times

Risk factors	Before DHS	During DHS	After DHS	Continuing DHS	Total
O2	0	0	0	18	18
Hypertension	405	1909	10	1405	3729
Diabetes	60	57	13	877	1007
Dyslipidemia	4	287	6	75	372
CKD	0	0	0	26	26
Atherosis	3	4	0	137	144
OSAS	0	0	0	1	1
Smoking	8	0	0	500	508
A2	9	0	0	86	95
FHCVD	0	0	0	10	10
Age	–	–	–	–	1859
Gender	–	–	–	–	1909

*DHS* duration of hospital stay, “–” denotes not considered

The experiment consists of two stages: the risk factor identification stage and the CVD prediction stage. In the first stage, we utilized all the labeled EMRs, including 830 in the training set, 119 in the development set, and 237 in the test set. In the second stage, we will extract the risk factors from the EMRs that need to be used to train the prediction model through the recognition model trained in the first stage. Among the EMRs utilized to train CVD prediction models, there are 461 training sets, 66 development sets, and 132 test sets. In the experiments, we used *Accuracy* (*A*), *Precision* (*P*), *Recall* (*R*), and *F-score* (*F*) as metrics for evaluating performance [18, 19]:

12 $$\begin{aligned} F=\frac{2*P*R}{P+R} \end{aligned}$$

### Models and parameters

As a comparison, we use different or in the case of ablation models for both stages of the experiment. In the models described next, the first two models are used in the risk factor identification stage, and the latter models are utilized for the CVD prediction stage.

*CRF* As a widely used traditional machine learning method, this model has been applied by Mao et al. [20] to the research of named entity recognition based on electronic medical records.

*BiLSTM-CRF* This model is a good example of combining deep learning with traditional machine learning methods. In the research on the automatic recognition of named entities in an extracted medical text, Li et al. [21] applied a model architecture that combines a bi-directional long short-term memory network (BiLSTM) and a conditional random field algorithm (CRF). The contextual information of sequences in the text is well encoded by BiLSTM and decoded using CRF.

*SVM* As one of the data mining techniques, support vector machine (SVM) is used by Menaria et al. [22] to support the research of medical decisions for correct diagnosis and treatment of diseases, and then explore to minimize the workload of experts.

*ConvNets* This model has a great influence in the field of text classification. Xiang et al. [23] proposed to represent each character with one-hot encoding and use a six-layer convolutional neural network to capture sequence information.

*LSTM/RFAB (no att)* As a special kind of recurrent neural network, Xin et al. [24] believes that it may be able to connect previous information with the current task and apply it in Alzheimer’s disease prediction research. On this basis, we construct a BiLSTM network model with one activation layer and one fully connected layer for CVD prediction. In fact, this is the RFAB model without the attention mechanism.

*RFAB* This model is proposed in this paper. In the experiments, we tune the hyperparameters by random search, and share all the experimentally selected hyperparameters as much as possible in Table 3.

**Table 3:** Hyper parameters of RFAB

Parameter	Description	Value
$d_w$	Dimension of word embedding	100
*lr*	Learning rate	le−3
*B*	Batch size	10
*dp*	Each neuron’s deactivation rate	0.5
*dr*	Decay rate for *lr*	0.99
*ds*	Number of decay steps	500
*h*	Each BiLSTM’s hidden unit quantity	256
*n*	Number of epochs	60

**Fig. 5:**
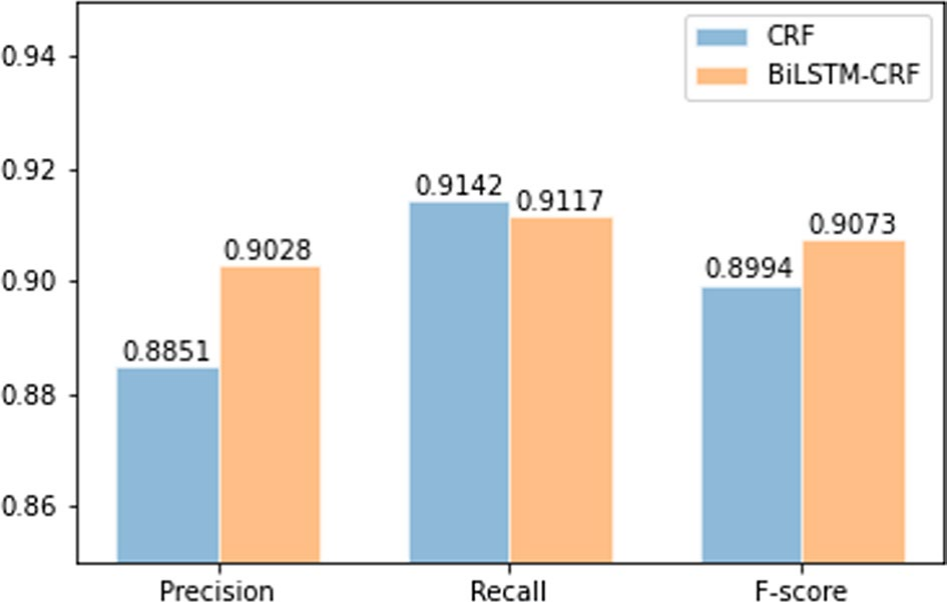
Comparison of CRF and BiLSTM-CRF models

### Experimental results

For the risk factor identification stage and cardiovascular disease prediction stage, we have carried out specific comparative experiments. In the second stage, we have done abundant experimental exploration from the aspects of input, embedding, and model ablation.

In Fig. 5, we compare the performance of CRF and BiLSTM-CRF on three evaluation indicators. On the whole, both of them perform well, but the latter’s F-score is better than the former. Therefore, we chose BiLSTM-CRF model as the risk factor extractor.

In Table 4, we show the comparison between the previous model and our proposed RFAB model for *Accuracy*, *Precision*, *Recall*, and *F-score*. And the performance of each model when the dataset is the original EMRs, the risk factor with the label, or the risk factor without the label. As shown in Figs. 1 and 2, the labels contain the corresponding category and time attributes for each risk factor. The $|\Delta |$ in the table represents the absolute value of the difference between the average of each model’s above four evaluation values and the best average.

In Table 5, we compared four cases: (1) The performance of the ConvNets model in random embedding. (2) The performance of the LSTM model in random embedding. (3) The performance of our model without attention mechanism, that is, the performance of the BiLSTM model. (4) When our model is in random embedding.

**Fig. 6:**
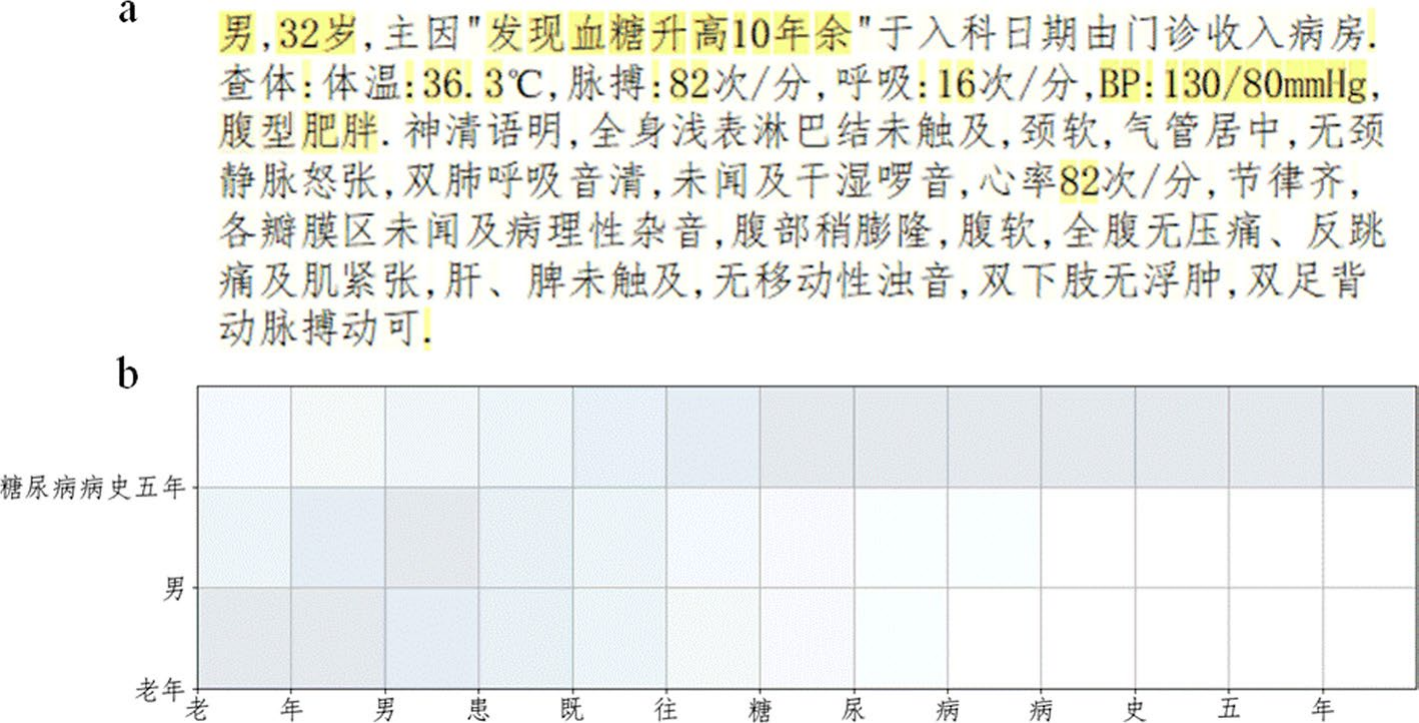
Visualization of learned attention *α*. **a** Basic physical status of a patient on EMR. **b** A description in the Case Characteristics module of a patient in EMR

In Fig. 6, we have made a visual example of the attention mechanism, which consists of the following two parts: (a) The specific conditions of the patient’s examination after entering the hospital in EMR. (b) A sentence from the case characteristics module in EMR. The label on the y-axis in the figure is a risk factor.

**Table 4:** The comparison of each model for CVD prediction results

Model	Accuracy %	Precision %	Recall %	F-score %	$|\Delta |$
$SVM_{(raw)}$	90.91	90.91	90.91	90.91	4.98
$SVM_{(no\ labels)}$	89.39	89.03	89.39	89.21	6.64
$ConvNets_{(raw)}$	92.83	92.64	92.83	92.73	3.13
$ConvNets_{(risks\ with\ labels)}$	93.94	89.43	93.21	91.28	3.93
$LSTM_{(raw)}$	92.24	93.46	92.73	93.09	3.01
$LSTM_{(risks\ with\ labels)}$	82.58	81.35	83.01	82.17	13.61
$RFAB_{(raw,\ no\ att)}$	93.91	93.83	93.91	93.86	2.01
$RFAB_{(risks\ with\ labels,\ no\ att)}$	89.23	88.96	89.23	89.07	6.77
$RFAB_{(no\ labels)}$	95.43	95.39	95.43	95.41	0.48
*RFAB*	95.87	95.98	95.87	95.86	–

**Table 5:** The performance of each model at random embedding

Model	Accuracy %	Precision %	Recall %	F-score %	$|\Delta |$
$ConvNets_{(risks\ with\ labels)}$	91.67	90.87	91.24	91.05	3.99
$LSTM_{(risks\ with\ labels)}$	81.82	79.45	82.36	80.88	14.07
$RFAB_{(no\ att)}$	92.31	92.18	92.31	94.09	2.47
*RFAB*	95.22	95.16	95.22	95.19	–

## Discussion

In Table 4, we compare the predictive performance of each model on different forms of corpora. When we utilize pre-trained embeddings along with labeled risk factors as the corpus, the RFAB model outperforms the other models on all four evaluation metrics. In addition, by comparing the performance of LSTM and BiLSTM models on the preprocessed raw EMRs and the labeled risk factor corpus, respectively, we can find that the prediction effect is not optimistic when the contextual information of the text sequence is not considered. Meanwhile, compared with the ConvNets proposed by Xiang et al. [23], we can find that the sequence models can better capture the contextual information of the EMR text than the convolutional neural network-based model. From the performance of RFAB in Tables 4 and 5, we can help improve the model’s accuracy in predicting CVD with the help of pre-trained character embeddings with a medical background. Moreover, from the performance of our model in Table 4 without attention mechanism, the significance of attention mechanism to the performance of this model is clearly reflected, and it also shows that the information of risk factors is more important to this prediction task. We exemplify the core part of the diagnostic basis in an electronic medical record based on the $\alpha$ learned by the attention mechanism in Fig. 6a. In addition, we also exemplify the associations between the three risk factors and their original character-level sequences in Fig. 6b. For Fig. 6, we emphasize that it can reduce the reading burden for doctors or individuals.

## Conclusions

Disease prediction research based on machine learning methods plays a pivotal role in supporting medical decisions for the correct diagnosis and treatment of diseases. Through the study of related technologies, doctors or individuals can quickly and accurately obtain key information and possible predictions after seeing a doctor, which is of great significance for reducing the pressure on experts and preventing diseases for individuals.

Aiming at the study of predicting CVD based on electronic medical records, this paper proposes an effective and reference idea to identify and extract risk factors and then rely on these key information to predict CVD. Meanwhile, we propose a corresponding CVD prediction model, a risk factor attention-based model (RFAB). With the help of the attention mechanism, the model effectively integrates the information between the risk factors and the context of the EMR text, and also considers the category and time attributes of the risk factors by the mean of labels. This enables the model to avoid redundant and confusing information, while focusing on effective key information, and can also take into account the original information of the EMR.

In the future, we will focus more on the research of CVD itself. Although the factors that can be found in EMRs that lead to CVD in individuals can be determined, it is undeniable that factors such as environment are diverse. Therefore, we will explore more comprehensive information sources, and then rely on machine learning methods to predict CVD efficiently and accurately.

## Data Availability

The datasets used and analyzed during the current study are available from the corresponding author upon reasonable requests. And, the related annotation resources are publicly available at https://github.com/nudt-nlp/RiskFactor.

## Abbreviations

CVD: Cardiovascular disease
RFAB: A risk factor attention-based model
LSTM: Long short-term memory
CRF: Conditional random field
BiLSTM: Bi-directional long short-term memory networks
BiLSTM-CRF: Bidirectional LSTM with a CRF layer
SVM: Support vector machine
ConvNets: Character-level convolutional networks
